# Automatic outer and inner breast tissue segmentation using multi-parametric MRI images of breast tumor patients

**DOI:** 10.1371/journal.pone.0190348

**Published:** 2018-01-10

**Authors:** Snekha Thakran, Subhajit Chatterjee, Meenakshi Singhal, Rakesh Kumar Gupta, Anup Singh

**Affiliations:** 1 Centre for Biomedical Engineering, Indian Institute of Technology Delhi, New Delhi, India; 2 Department of Computer Science and Engineering, Indian Institute of Technology Delhi, New Delhi, India; 3 Department of Radiology, Fortis Memorial Research Institute, Gurgaon, India; 4 Department of Biomedical Engineering, All India Institute of Medical Sciences Delhi, New Delhi, India; University of Chicago, UNITED STATES

## Abstract

The objectives of the study were to develop a framework for automatic outer and inner breast tissue segmentation using multi-parametric MRI images of the breast tumor patients; and to perform breast density and tumor tissue analysis. MRI of the breast was performed on 30 patients at 3T-MRI. T_1_, T_2_ and PD-weighted(W) images, with and without fat saturation(WWFS), and dynamic-contrast-enhanced(DCE)-MRI data were acquired. The proposed automatic segmentation approach was performed in two steps. In step-1, outer segmentation of breast tissue from rest of body parts was performed on structural images (T_2_-W/T_1_-W/PD-W without fat saturation images) using automatic landmarks detection technique based on operations like profile screening, Otsu thresholding, morphological operations and empirical observation. In step-2, inner segmentation of breast tissue into fibro-glandular(FG), fatty and tumor tissue was performed. For validation of breast tissue segmentation, manual segmentation was carried out by two radiologists and similarity coefficients(Dice and Jaccard) were computed for outer as well as inner tissues. FG density and tumor volume were also computed and analyzed. The proposed outer and inner segmentation approach worked well for all the subjects and was validated by two radiologists. The average Dice and Jaccard coefficients value for outer segmentation using T_2_-W images, obtained by two radiologists, were 0.977 and 0.951 respectively. These coefficient values for FG tissue were 0.915 and 0.875 respectively whereas for tumor tissue, values were 0.968 and 0.95 respectively. The volume of segmented tumor ranged over 2.1 cm3–7.08 cm^3^. The proposed approach provided automatic outer and inner breast tissue segmentation, which enables automatic calculations of breast tissue density and tumor volume. This is a complete framework for outer and inner breast segmentation method for all structural images.

## Introduction

Magnetic Resonance Imaging (MRI) is a widely used technique in breast cancer diagnosis, stage identification and monitoring of treatment responses. In general, an MRI image of the breast also includes other body parts (such as lung, heart, liver, pectoral muscle) and separation of breast tissue from rest of the body part is often required for further analysis. Breast tissue itself is composed of different components particularly, fibro-glandular (FG) and fatty tissues. In the patient data, tumor/lesion can be another component of breast tissue. Segmentation of these breast components is required for breast density estimation, post-treatment evaluation of neoadjuvant chemotherapy or chemoprevention process [1–3] and in tumor localization during radiotherapy treatment. It has been reported that women with high level of FG tissue are more prone to cancer [4–6].

Automatic segmentation of breast tissue reduces subjectivity on results and can speed up the data processing. Automatic segmentation of breast tissue in MRI image is a two-step process. In the 1^st^ step, breast area has to be separated from chest wall including pectoral muscle, which is termed as outer breast segmentation and in the 2^nd^ step inner segmentation of breast tissue into FG, fatty and tumor tissue is performed. Automatic segmentation of breast tissue is a challenging task due to large variations in breast sizes and shapes, intensity inhomogeneity, image artifacts and other noise errors [7,8].

Manual segmentation of breast tissue is tedious and it is impractical to segment entire breast tissue involving a large number of diverse data sets. Moreover, manual segmentation of inner breast tissue might not be accurate due to heterogeneous nature of tissues like FG. A number of semi-automatic breast tissue segmentation approaches have been reported [9–14]. However, these approaches require some user input and are suitable in segmenting only specific part of the breast tissue. Some automatic breast tissue segmentation approaches based on supervised and unsupervised learning have been reported in the literature [15–36]. Some unsupervised methods like active contour [37], hessian filtering [21,22], fuzzy c-mean clustering [16,17,29,38] are not fully automatic as they require manually segmented breast region as input to find further breast tissues (inner). However, they were not included into a complete automatic framework for the analysis of breast density. A number of supervised approaches have also been reported like template based [39], atlas-based approach [34] etc. Some of these approaches require manually segmented training datasets (e.g., the atlas-based approaches) or have been proposed for particular data sequence. Template-based methods require the construction of a template data. Due to the large variability in the shape of breasts, simply using one universal template may not be robust enough to segment all types of breasts.

Reported methods are limited to providing segmentation of either outer or one of the inner tissue only. Most of these reported segmentation methods for breast segmentation were based upon single MRI image. A single MRI image of breast might not be sufficient for segmenting accurately all tissues of the breast, particularly inner tissues. In general, MRI protocol includes different types of MRI data, such as T_1_-weighted(W), T_2_-W, PD-W, Perfusion-W images etc. Multi-parametric MRI images can be used for segmentation of different tissues. Recently, a SVM-based approach using multi-parametric MRI images [35] was proposed for inner segmentation of breast tissue. In this approach, T_1_-W, T_2_-W and PD-W and three-point dixon water-only and fat-only contrasts images were included. This approach works well for fatty and FG tissue segmentation; however, it might fail to segment tumor/lesion in case of similar signal intensity between tumor and FG tissue on T_2_-W images. Outer segmentation of breast MRI is a challenging problem mainly due to large variations in tissue contrast of different MRI images and lack of standardized protocols. Most of the reported outer segmentation methods might not work for all types of structural images (T_1_-W/T_2_-W/PD-W) but these structural modalities are equally important in clinical aspects. So the motivation behind this work regarding outer segmentation is to make the algorithm more robust for all structural images.

The objective of this study was to use multi-parametric MRI images (any of structural images (T_1_-W/T_2_-W/PD-W) with and without fat saturation (WWFS) and difference image of pre and post contrast dynamic contrast-enhanced (DCE) MRI data), for developing an automatic framework to segment breast into outer as well as inner tissues such as FG, fatty and tumor. The performance of the automatic segmentation methods against manual segmentation is evaluated using the similarity coefficients.

## Materials and methods

In this study, we have included breast MRI data of 30 female patients (25–80 years) scanned in the hospital (Fortis Memorial Research Institute, Gurgaon, India) during 2015–2016. All the subjects had satisfied inclusion criteria of referral to this hospital for MRI scanning. MRI study was pre-approved by the Institutional Review Board of the hospital (Fortis Memorial Research Institute, Gurgaon, India) and written informed consent was taken before MRI scanning. Patients were suspected of breast lesions on mammography and/or USG and were sent for further evaluation for contrast-enhanced MRI.

### MRI data acquisition

All the MRI experiments were performed at 3T whole body Inginia MRI system (Philips Healthcare, The Netherlands) with the help of a 7-channel biopsy compatible breast coil. After a localizer, 2D T_1_-W, T_2_-W and PD-W images, WWFS, were acquired using turbo spin echo (TSE) pulse sequence followed by 3-D DCE-MRI (with fat suppression) data acquisition in the axial orientation. Fat suppression was based upon two-point modified DIXON (m-DIXON) TSE approach [40,41]. In this study, we have referred ‘In Phase’ images as without fat suppression and reconstructed water only images as with fat suppression. Gd-BOPTA (Multihance, Bracco, Italy) in a dose of 0.1 mmol/kg body weights was administered intravenously with the help of a power injector at a rate of 3.0 mL/sec, followed by a bolus injection of a 30-mL saline flush. Forty time points were acquired with a temporal resolution approximately of 5.4 seconds for each time point. The details of MRI parameters are shown in Table 1.

**Table 1:** MRI protocols parameters.

Clinical Setting	DCE-MRI	T_2_-W	T_1_-W	PD-W
**Orientation**	Axial	Axial	Axial	Axial
**TR/TE(ms/ms)**	3/1.5	2974/100	603/10	2974/30
**Flip angle(deg)**	12	90	90	90
**Field of view(mm**^**2**^**)**	338*338	338*338	338*338	338*338
**Slice thickness(mm)**	3	3	3	3
**No of Slices**	60	60	60	60
**Acquisition matrix size**	228*226	452*338	452*338	452*338
**Interpolated matrix size**	512*512	512*512	512*512	512*512
**Pulse sequence**	Fast field echo	Turbo spin echo	Turbo spin echo	Turbo spin echo
**Acquisition mode**	3D	2D	2D	2D

### Data processing

Data were processed using in-house developed routines in MATLAB 2013a (The MathWorks Inc., Natick, MA, USA 2013). Various data processing steps are described below:

#### Co-registration

In the current study, there were small (<2mm) rigid body motions between different types of MRI images of same section/volume. Co-registration was performed using ‘imregister’ routine in MATLAB to correct misalignment between the multiple scans. T_2_-W images were taken as reference images for registration (Multimodal optimization parameters: Optimizer: one-plus-one evolutionary; metric: Mattes Mutual Information; Growth factor: 1.02; Epsilon: 1.500000e-06; Initial radius: 0.006; Maximum iterations: 500).

#### Manual segmentation procedure

**Fig 1:**
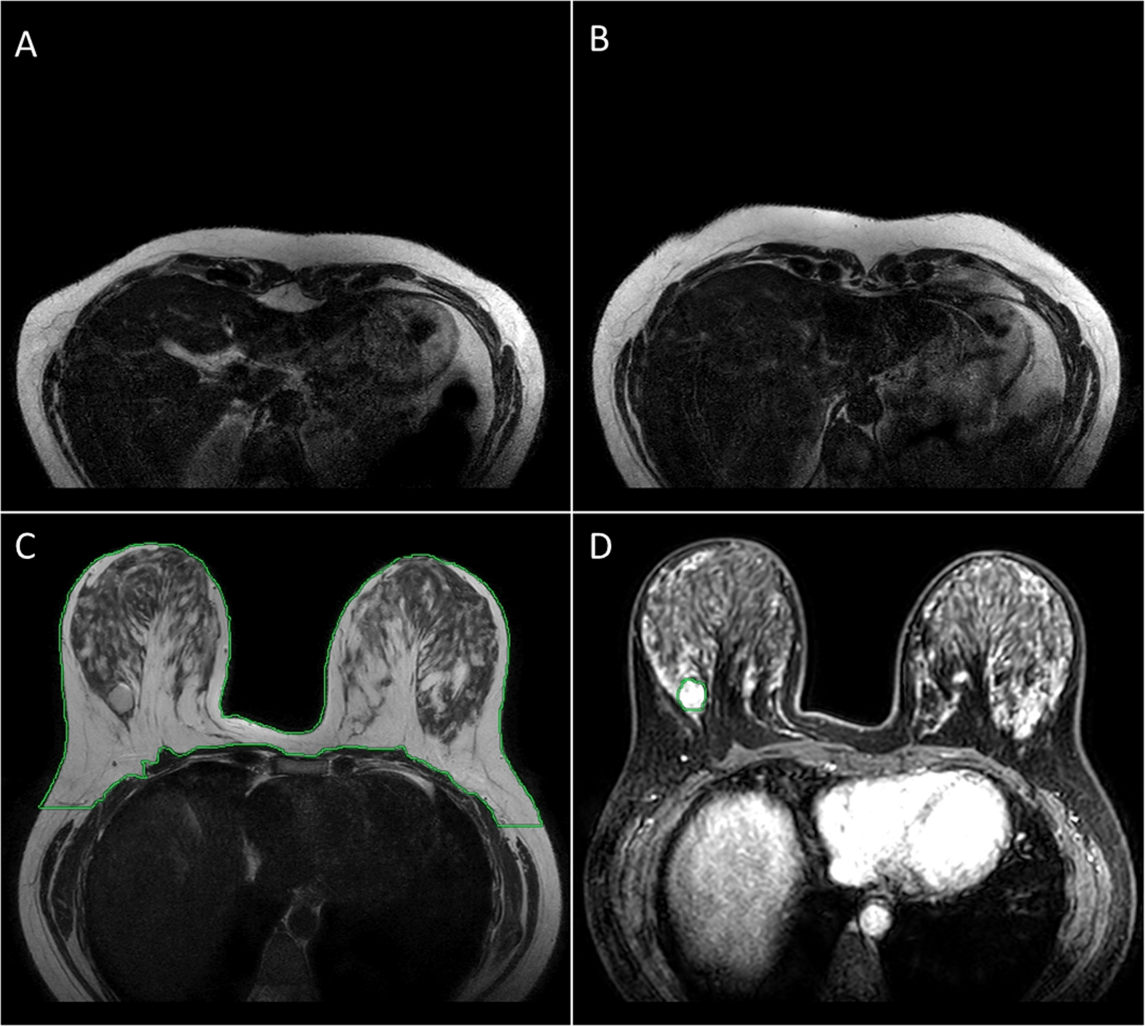
Manual segmentation. (A and B) show Extreme Slices (C) ROI was drawn on T_2_-W without fat saturation images for separating out the breast tissue. (D) ROI was drawn on tumor tissue. Dark green color shows the ROI contour on both images.

Manual segmentation was performed by two experienced radiologists using RadiAnt DICOM Viewer 3.4.2 (http://www.radiantviewer.com/). Manual segmentation of outer breast tissue was carried out on every third slice of T_2_-W, T_1_-W and PD-W images without fat saturation. For example, manual segmentation on the T_2_-W image is shown in Fig 1C. In the next step, an ROI was drawn on tumor slices of DCE-MRI images (Fig 1D). The FG manual segmentation was done on those datasets which have well-connected FG structure as well as clear visible boundaries. Image slices on which significant breast tissues are not clearly visible as shown Fig 1A and 1B are called extreme slices.

#### Automatic outer segmentation algorithm

**Fig 2:**
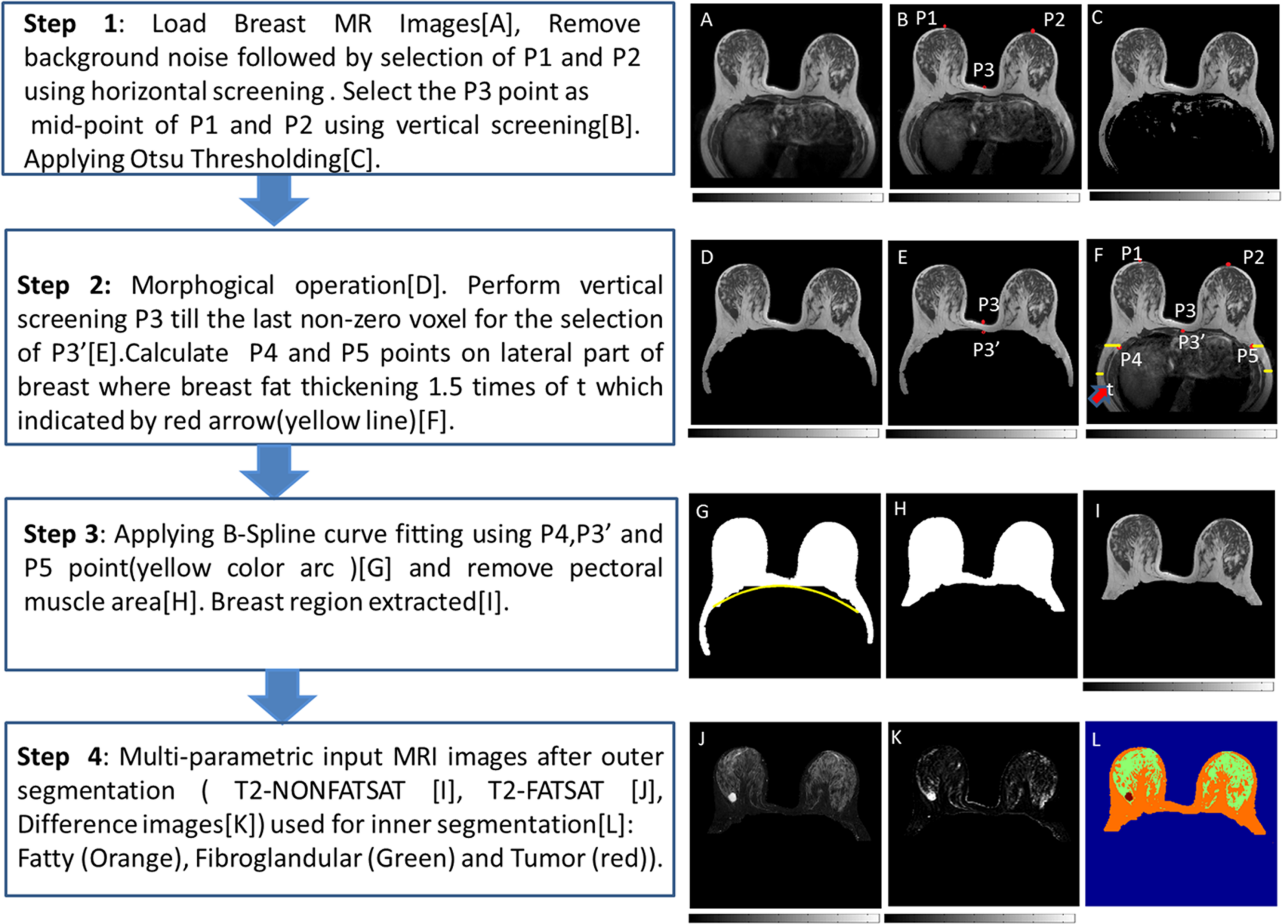
General overview of the process for outer and inner segmentation in breast MR images. (A) Original T_2_-W MRI Image. (B) Image shows points P1, P2 and P3. (C) Image after fractional Otsu thresholding. (D) Image after the morphological operation. (E) Image shows points P3 and P3’(new P3 point). (F) Image shows points P1, P2, P3, P3’, P4, P5 and fat thickness t, indicated by red arrow. (G) Image shows an overlay of the fitted B-spline curve (yellow) through P4, P3’ and P5. (H) Outer segmented mask image. (I) Outer segmented breast tissue overlaid on the original T_2_-W image using the proposed method. (I) and (J) show Non-Fat-Sat T_2_-W and Fat-Sat T_2_-W images after outer segmentation respectively. (K) Difference image. (L) Inner segmentation image in which FG indicated by green color, fatty tissue by orange and tumor by red color.

The steps of automatic outer segmentation are described below:

Step 1: Pre-processing was performed on structural images (T_2_-W/T_1_-W/PD-W) for background noise removal. In the current study, we used 5% of the maximum signal intensity of the center slice as threshold value followed by morphological operation opening (kernel size = 3). The proposed algorithm can work for all the slices but the user can select relevant slices (remove the extreme slices automatically) for the processing in order to avoid irrelevant computations.
Step 2: In this step, landmark points P1, P2, P3 were automatically selected on each slice of breast MRI data. Points P1 and P2 were obtained by automatic screening of profiles of signal intensity of horizontal lines from left side to mid-point and right side to mid-point of the image respectively. Pixels with first non-zero value were named as P1 and P2 respectively. The first non-zero pixel on the vertical line passing through mid-point of P1 and P2 was termed as P3 (Fig 2B). Breast MRI image was divided into two parts (upper and lower) using the horizontal line through point P3.

Step 3: The lower part of the breast image mainly contains other body parts (liver, heart etc.) and some part of the breast. To segment out those parts, fractional Otsu thresholding (Fig 2C) was applied only to non-zero pixels of the lower part followed by morphological operations (erosion and dilation with a kernel size of 7 and filling hole operations (Fig 2D). The upper part was kept untouched in this step. These two parts were merged back for further processing in the next step.
Step 4: Main goal of this step was to refine the selection of point P3 to P3’ and to find out corner points P4 and P5. For the selection of point P3’, vertical screening was done from P3 till the last non-zero pixel (shown in Fig 2E). On the basis of anatomical observation, it was found that two extreme endpoints can be obtained while screening from top left or top right or to bottom left or bottom right and the yellow line specifies the width of fat measured from the above calculated endpoints in left and right breast toward inside. Now the points P4 and P5 were marked when fat width increases to 1.5 times than the width of the yellow line which is indicated by red arrow (shown in Fig 2F). The factor 1.5 was based on empirical observation and validated by radiologists. Points P4, P5 were calculated from a middle slice of breast image and kept fixed for other slices. P3’ point was calculated for each slice (Fig 2F).
Step 5: It could be possible that some non-breast part might be left even after applying fractional otsu thresholding and morphological operations in Step-3. Therefore, the B-spline curve was fitted using three points P4, P3’ and P5 on breast image after step-3. This was followed by setting the values of pixels below the fitted curve to zero as shown in Fig 2G. Part of the image below a horizontal line through points P4 and P5 towards the bottom edge respectively was also removed.
Step 6: For removal of any leftover pectoral muscle, fractional otsu thresholding followed by morphological operation opening (kernel size = 3), was applied on pixels with non-zero intensity values below mid-point of P3 and P3’. This step results in the final mask as shown in Fig 2H. Finally, mask overlaid on the original image is shown in Fig 2I.

This algorithm was repeated for T_1_-W, T_2_-W and PD-W respectively but we present the whole process on T_2_-W image for simplicity.

#### Automatic inner segmentation algorithm

Inner segmentation of breast tissue was achieved using different types of MR images. Process for inner segmentation is described below:

Step 1: Outer segmentation of breast tissue from rest of body parts (mask1) as shown in Fig 2H.
Step 2: Subtraction of images (T_2_-W/T_1_-W/PD-W) with fat suppression from without fat suppression followed by Otsu threshold operation resulted in fat tissues mask (mask2).
Step 3: Subtraction of mask-2 from mask-1 provided a mask (mask-3) for FG and tumor.
Step 4: A separate tumor/lesion mask was generated using difference image/stack (DI) images followed by Otsu thresholding and morphological operation (erosion and dilation with disc size of 9) (mask-4). DI was computed using post and pre-contrast DCE-MRI images followed by normalization (using pre-contrast DCE-MRI images). In order to reduce noise effect, the pre-contrast image was taken as the average of first four time points and the post-contrast image was taken as maximally enhanced time point in DCE series and take average with three more time points around the maximum time point’s neighborhood. (Fig 2K).
Step 5: Segmentation of FG tissue was obtained given by as mask5 = (mask3-mask4).

Inner segmentation algorithm provided mask2, mask4 and mask5 for fat, tumor, and FG tissue respectively. A combined mask, with separate values for fat, FG and tumor tissue was also generated (Fig 2L). Skin tissue was removed using hessian based sheet-ness filter method [22].

The volume of segmented tissues, particularly breast density and tumor were calculated. 3-D reconstruction of breast tissue, without and with segmentation, were generated using Volume viewer plugin of ImageJ [42].

### Statistical analysis

The performance of a proposed method for outer segmentation and inner segmentation were tested using similarity coefficients such as Dice coefficient(DSC) [43] and Jaccard coefficient [44]. For this purpose, manual segmentation was done by two expert radiologists. Additionally, we determined Sensitivity index [45]. FG density and tumor volume were computed for each patient. Additionally, separate volumes and density for left and right breast were computed. The Dice coefficient and Jaccard coefficient metrics are given by following equations:

Dicecoefficientd=(2×|A∩B|)/(|A|+|B|) (1)

Jaccardcoefficientp=(|A∩B|)/(|A|+|B|−|A∩B|) (2)

Where A is the manual segmentation by expert radiologist and B is the automatic segmentation. Udupa et al.[45] described sensitivity which is defined as the intersection between A (Manual) and B (Automated) divided by automated segmentation (B).

Additionally, Sensitivity parameter is computed as follows:

Sensitivitys=(|A∩B|)/(|B|) (3)

Percentage of breast density is defined as:

Breastdensity(%)BD=(TotalFGvolume×100)/Totalbreastvolume (4)

Mean and standard deviation (SD) of similarity coefficients for all the patient datasets were calculated. While calculating Dice and Jaccard coefficients, every third slices were considered for both automatic and manual segmentation of outer and FG segmentation. Breast tumor tissue was not considered while calculating breast density. Bland-Altman plots[46] were used for comparing breast density of left (L) and right (R) breast. The plot was computed as: [X, Y] = [(L+R)/2, (L-R)]. In addition, the correlation coefficient was used to evaluate the significance of the correlation between left (L) and right (R) breast density. Average density for different age groups (20–40, 40–60, 60–80 years) was also computed.

## Results

In the current study, the proposed method was applied on 30 patient datasets and validated by two experienced radiologists. Both outer and inner segmentation worked well for all subjects. In the current study, there were small (<2mm) rigid body motions between different types of MRI images of same section/volume. These motions were mitigated by MATLAB inbuilt registration algorithm in the pre-processing step.

**Fig 3:**
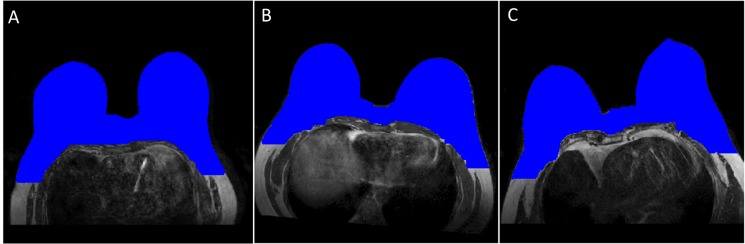
Proposed method results for outer segmentation. It shows outer breast segmentation results of different patients with different shapes and sizes with different positions. Segmented images of breast tissue were color overlaid on base gray scale T_2_-W images. Blue color shows outer segmentation.

**Fig 4:**
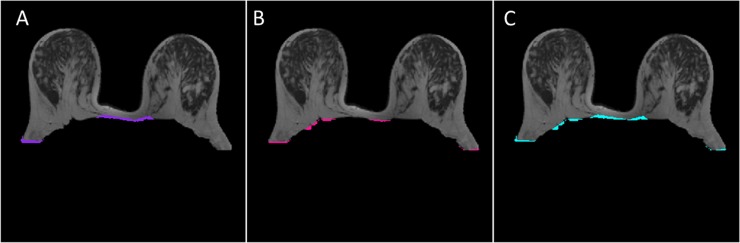
Example of overlapping results for outer segmentation. (A) The region of difference images between manual-1 and automatic proposed method is shown by purple color whereas (B) manual-2 and automatic proposed method is shown by pink color. The region of difference between two manual segmentations is shown by cyan color(C).

Results of breast segmentation for different patients with different breast shapes and sizes with different positions using proposed outer segmentation method are shown in Fig 3. Manual outer segmentation was carried out by two experienced radiologists. The region of difference between three cases on T_2_-W images (case 1: manual-1 vs automatic, case 2: manual-2 vs automatic and case 3: manual-1 vs manual-2) are shown in Fig 4. It provided accurate result except having 2–3% mismatch in the lateral part of the breast. Purple, pink and cyan colors show the region of difference.

**Fig 5:**
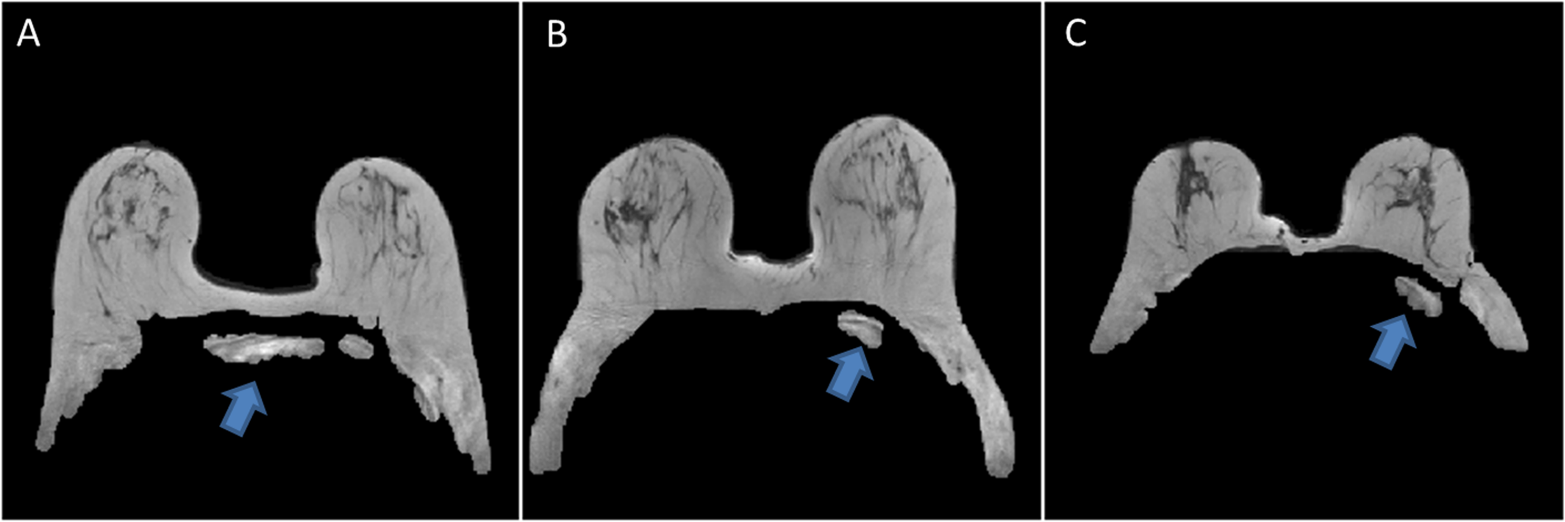
Example of different datasets after the morphological operation. Segmented images show an example of leftover points (non-breast tissue part) which were removed after B-spline curve fitting. Arrows indicate non-breast tissue part.

In some cases after otsu thresholding and morphological operations, some non-breast tissue portions were left after P3’ point as shown in Fig 5A–5C. These leftover point were removed by B-spline fit using P4, P3’ and P5 points. Arrows indicate non breast tissue part.

**Fig 6:**
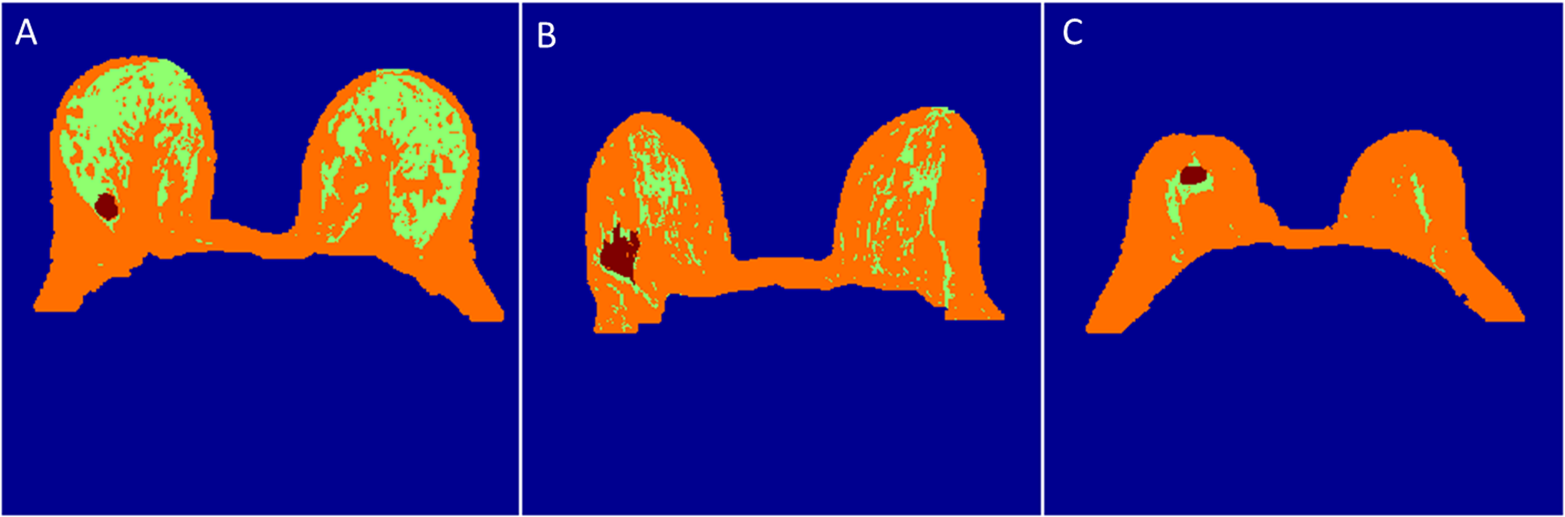
Inner segmentation results. It shows inner segmentation of breast tissue into FG (green color), fatty (orange color) and tumor (red color) tissue for a single slice of different patients.

Inner segmentation of breast tissue using multi-parametric MRI images also worked well and breast tissue was segmented out into FG, fatty and tumor tissues. Inner breast segmentation results for a single slice of different patients in which FG tissue indicated by green color, fatty by orange color and tumor by red color are shown in Fig 6.

**Fig 7:**
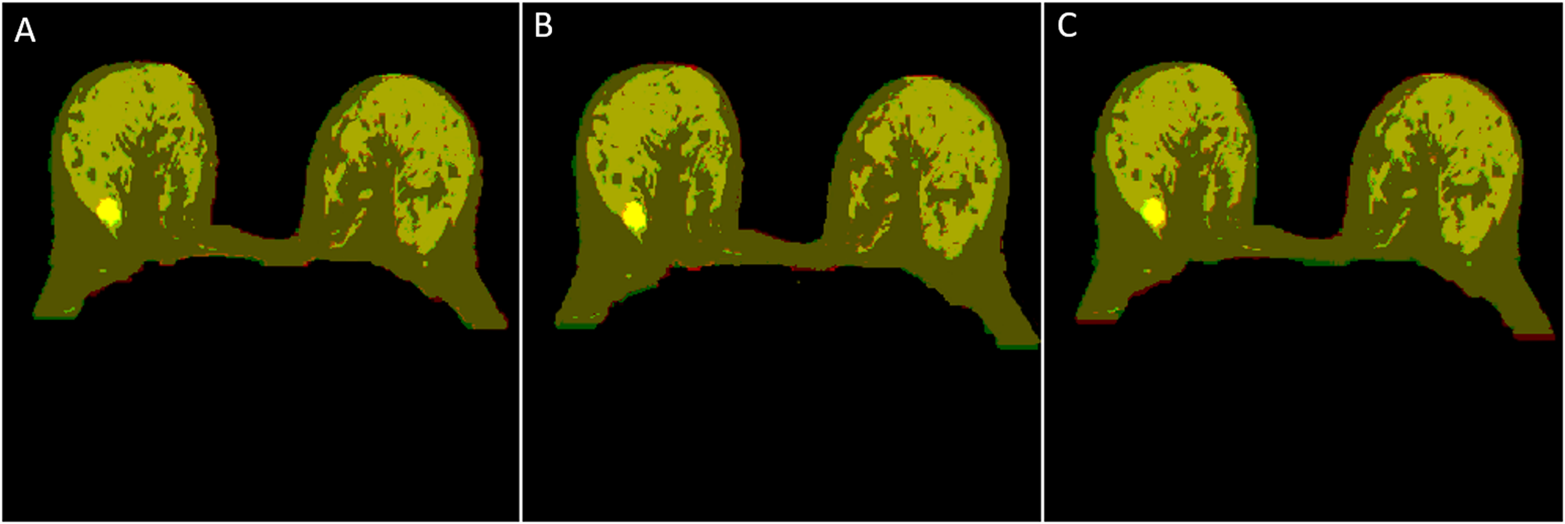
Comparison between different structural MRI images. **(**A), (B) and (C) show T_1_-W vs T_2_-W, PD-W vs T_2_-W and PD-W vs T_1_-W respectively. Yellow shades show overlapping between two mask whereas red and green color for present in one mask but not other mask respectively.

Outer and FG segmentation method worked well for T_2_-W, T_1_-W and PD-W images. The overlapping between structural MRI images (T_1_-W vs T_2_-W, PD-W vs T_2_-W and PD-W vs T_1_-W) seem significant. It provided similar result except having 3–5% mismatch in the lateral part whereas 5–8% mismatch in the inner part of the breast. Red and green colors show the region of difference whereas yellow shades show overlapping region between two mask as shown in Fig 7.

**Fig 8:**
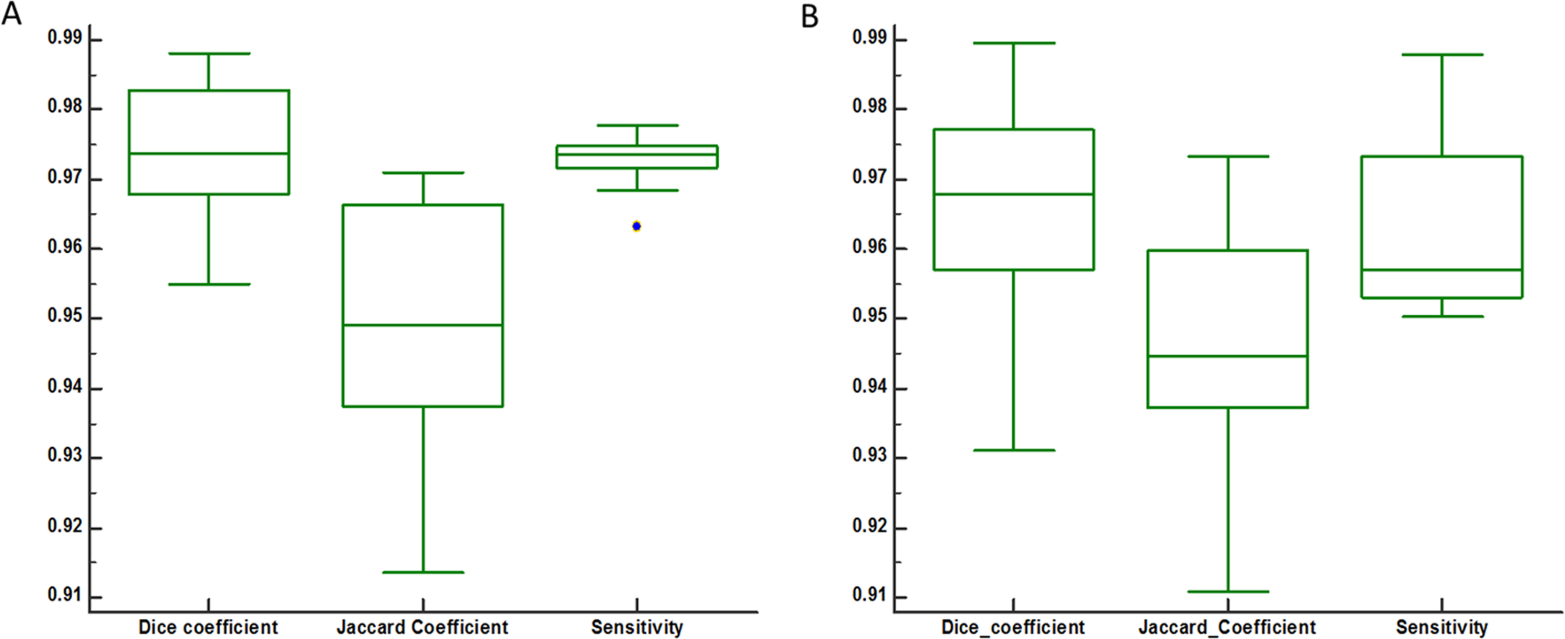
Performance of proposed method for outer segmentation and inner (tumor) segmentation evaluated on the basis of similarity index obtained by one radiologist. It shows the distribution of three indexes Dice, Jaccard and Sensitivity coefficient for outer breast (A) and tumor tissue (B).

Table 2 shows the average value of DSC, Jaccard, and Sensitivity index for 30 patients. Column 6 shows DSC, Jaccard and Sensitivity index of outer segmentation obtained by two radiologists whereas column 4 and column 5 show manual-1 vs automatic and manual-2 vs automatic respectively. The average DSC and Sensitivity values for outer segmentation using T_2_-W, obtained by two radiologists, were both above 0.977 whereas Jaccard coefficient was 0.951. The similarity coefficients obtained by a radiologist are also shown in Fig 8A. The accuracy of outer segmentation results obtained using T_1_-W and PD-W images were similar to those obtained using T_2_-W images as shown in Table 2.

**Table 2:** Dice coefficient, Jaccard coefficient and sensitivity for outer segmentation.

S. No	Similarity Index	Structural MRI	Radiologist 1 Manual-1 vs Automatic Outer Segmentation (Mean±SD)	Radiologist 2 Manual-2 vs Automatic Outer Segmentation (Mean±SD)	Inter-radiologist Radiologist 1 vs Radiologist 2 Outer Segmentation (Mean±SD)
1	DSC	T_2_-W	0.973±0.010	0.982±0.012	0.989±0.010
		T_1_-W	0.962±0.012	0.971±0.009	0.982±0.013
		PD-W	0.957±0.030	0.951±0.010	0.991±0.009
2	JACCARD	T_2_-W	0.948±0.018	0.954±0.015	0.993±0.011
		T_1_-W	0.933±0.020	0.940±0.011	0.989±0.012
		PD-W	0.928±0.021	0.930±0.010	0.996±0.009
3	SENSITIVITY	T_2_-W	0.970±0.007	0.972±0.008	0.998±0.009
		T_1_-W	0.967±0.005	0.969±0.006	0.997±0.005
		PD-W	0.951±0.003	0.948±0.010	0.995±0.004

Mean and standard deviation (SD) values of Dice coefficient (DSC), Jaccard coefficient and Sensitivity between manual and automatic segmentation for outer breast tissue from 30 different patients.

Tumor tissue was also segmented well as demonstrated by high values of similarity coefficient shown in Fig 8B. Table 3 shows the average value of DSC, Jaccard, and Sensitivity index for tumor segmentation of DCE-MRI. Column 6 shows DSC, Jaccard coefficient and Sensitivity of tumor segmentation obtained by two radiologists whereas column 4 and column 5 show manual-1 vs automatic and manual-2 vs automatic respectively. The average DSC and Sensitivity values for tumor segmentation, obtained by two radiologists for DCE-MRI images, were both above 0.968 whereas Jaccard coefficient was 0.95.

**Table 3:** Dice coefficient, Jaccard coefficient and sensitivity for tumor segmentation.

S. No	Similarity Index	Radiologist 1 Manual-1 vs Automatic Tumor Segmentation (Mean±SD)	Radiologist 2 Manual-2 vs Automatic Tumor Segmentation (Mean±SD)	Inter-radiologist Radiologist 1 vs Radiologist 2 Tumor Segmentation (Mean±SD)
1	DSC	0.966±0.015	0.970±0.013	0.989±0.014
2	JACCARD	0.947±0.016	0.953±0.018	0.991±0.016
3	SENSITIVITY	0.962±0.012	0.969±0.009	0.987±0.010

**Fig 9:**
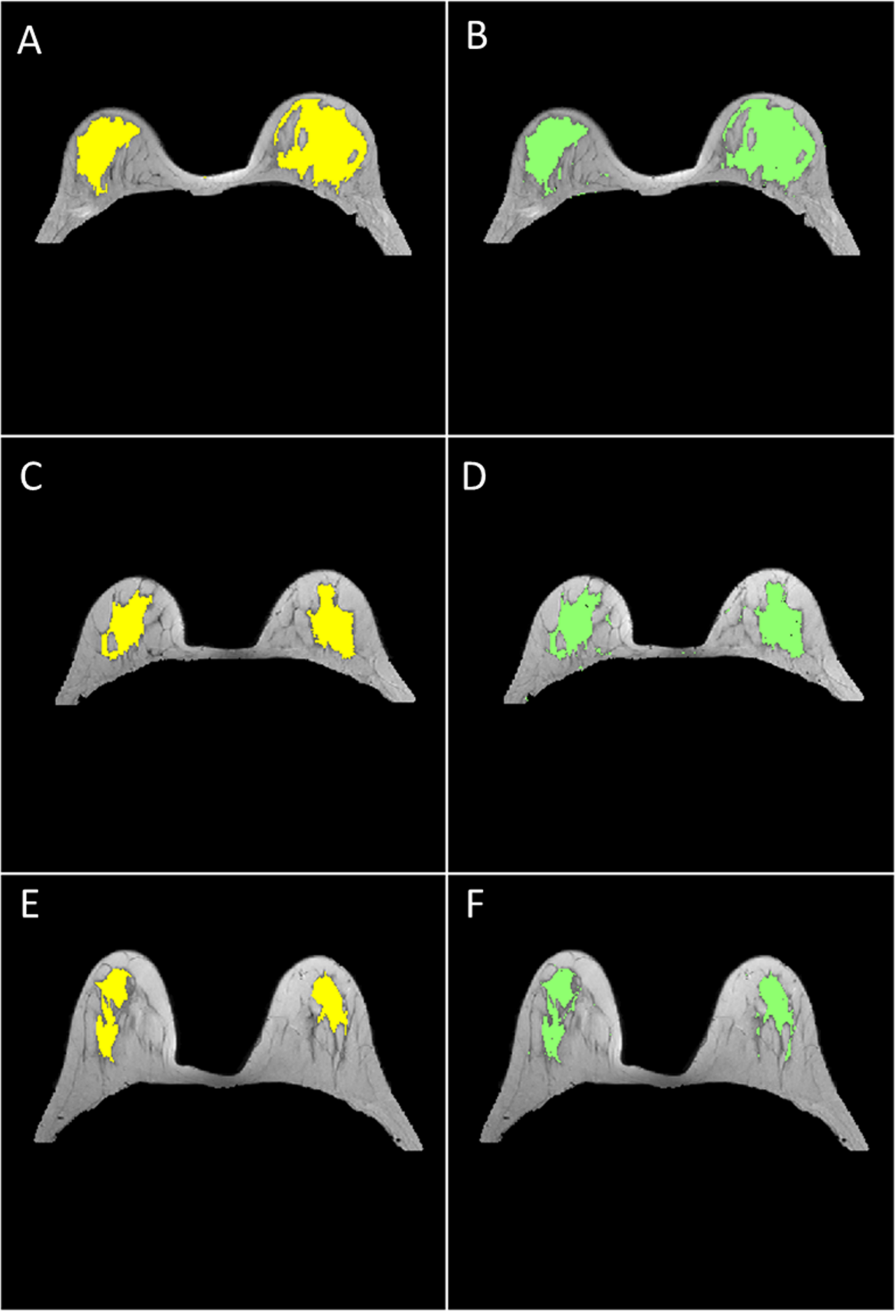
FG segmentation results. Fig 9A, 9C and 9E show manual FG segmentation by a radiologist which is indicated by yellow. Fig 9B, 9D and 9F show automatic FG segmentation which is indicated by green color.

Out of 30 datasets, we have found 11 datasets which have well-connected FG structure as well as clear visible boundaries as shown in Fig 9. These datasets were manually segmented by two expert radiologists. In Fig 9, yellow color indicates manual segmentation whereas green color shows automatic segmentation of FG tissue. For rest 19 datasets, FG tissue contains many thin structures or patterns, which were difficult to segment manually by radiologists. Therefore, it was difficult to validate the results with manual segmentations.

Table 4 shows the average value of DSC, Jaccard, and Sensitivity index for FG tissue segmentation of T_2_-W, T_1_-W and PD-W for 11 patients. Column 6 shows DSC, Jaccard and Sensitivity index of FG segmentation obtained by two radiologists whereas column 4 and column 5 show manual-1 vs automatic and manual-2 vs automatic respectively. The average DSC and Sensitivity values for FG segmentation using T_2_-W, obtained by two radiologists, were both above 0.915 whereas Jaccard coefficient was 0.875. The accuracy of FG segmentation results obtained using T_1-_W and PD-W images were similar to those obtained using T_2_-W images as shown in Table 4.

**Table 4:** Dice coefficient, Jaccard coefficient and sensitivity for FG segmentation.

S. No	Similarity Index	Structural MRI	Radiologist 1 Manual-1 vs Automatic FG Segmentation (Mean±SD)	Radiologist 2 Manual-2 vs Automatic FG Segmentation (Mean±SD)	Inter-radiologist Radiologist 1 vs Radiologist 2 FG Segmentation (Mean±SD)
1	DSC	T_2_-W	0.92±0.04	0.91±0.03	0.97±0.02
		T_1_-W	0.90±0.02	0.89±0.01	0.96±0.04
		PD-W	0.86±0.04	0.87±0.02	0.94±0.05
2	JACCARD	T_2_-W	0.88±0.03	0.87±0.04	0.98±0.04
		T_1_-W	0.84±0.01	0.84±0.02	0.98±0.02
		PD-W	0.81±0.04	0.79±0.03	0.96±0.06
3	SENSITIVITY	T_2_-W	0.91±0.05	0.93±0.02	0.96±0.03
		T_1_-W	0.89±0.02	0.90±0.04	0.98±0.02
		PD-W	0.83±0.03	0.81±0.01	0.97±0.03

**Fig 10:**
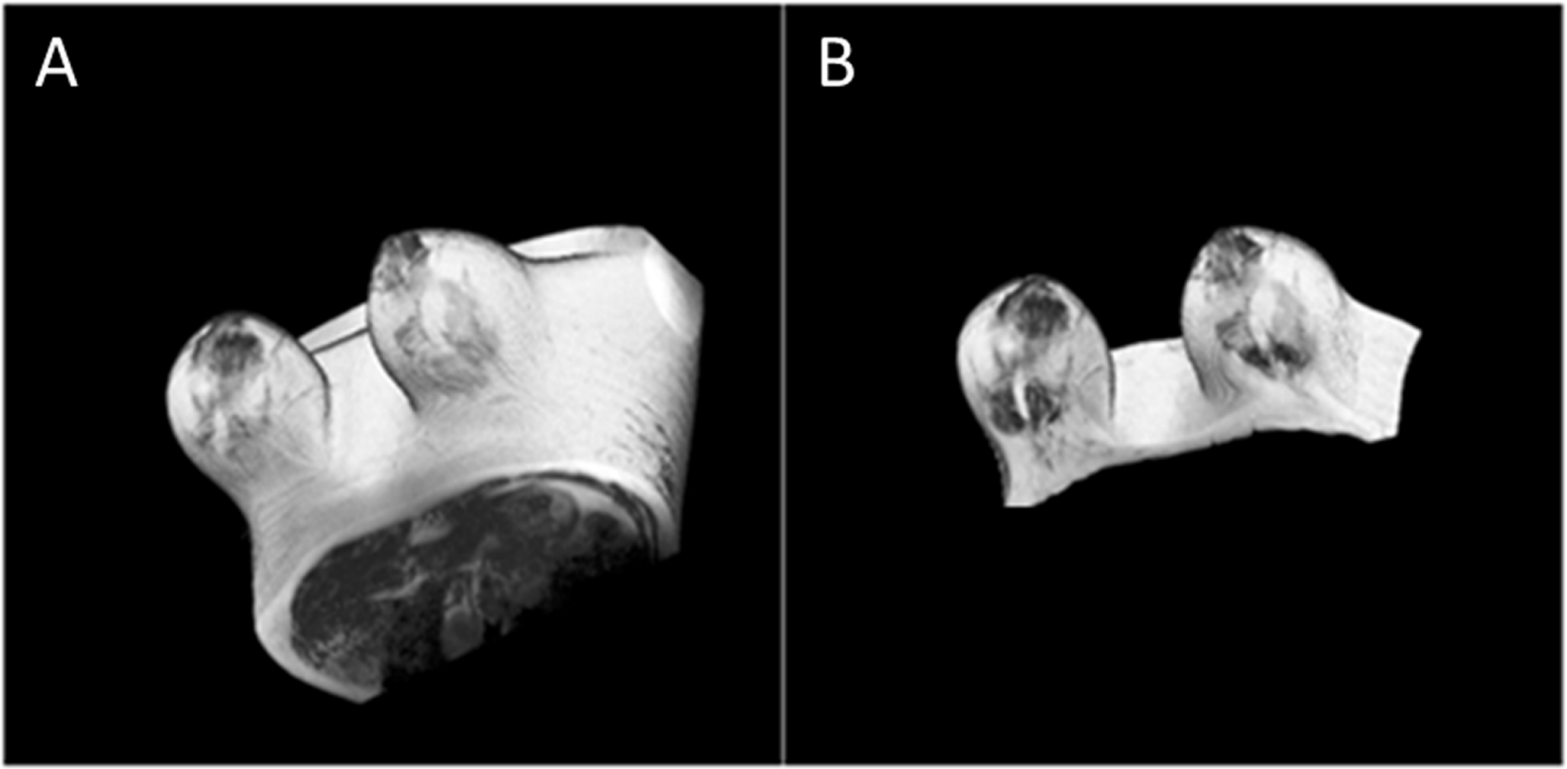
Example for the whole breast as 3D visualization before and after outer segmentation. (A) shows a 3D visualization of T_2_-W Image before outer segmentation whereas (B) shows after outer segmentation.

The 3-D view of the entire breast before and after outer segmentation (using volume viewer plugin of ImageJ) is shown in Fig 10.

Total breast volume, FG tissue volume and tumor volume were calculated for each patient dataset. The mean value of total breast volume and FG volume in both sides were 1526 cm^3^ and 124 cm^3^ respectively. In the current study, breast density ranged from 2.2%-27% over different subjects. The mean value of breast density was 8.6% as shown in Table 5.

**Table 5:** The FG tissue volume segmented within the breast region was obtained using the proposed method. Total breast volume (cm^3^), FG tissue volume (cm^3^) and percentage of breast density were calculated. Tumor volume (cm^3^) was also calculated for 28 datasets.

S. No	Total Breast Volume(cm^3^)	FG Volume (cm^3^)	Breast Density (%)	Tumor Volume(cm^3^)
1	1986.64	44.49	2.24	4.47
2	1779.41	363.42	20.42	2.32
3	1444.67	346.17	23.96	2.47
4	1601.20	95.35	5.95	5.62
5	1420.03	163.24	11.50	7.08
6	1635.72	96.56	5.90	4.19
7	1639.31	128.79	7.86	3.33
8	1382.35	69.68	5.04	4.84
9	1718.16	106.23	6.18	3.26
10	1669.79	94.98	5.69	5.05
11	430.43	72.53	16.85	2.02
12	1571.56	99.63	6.34	4.07
13	1716.63	100.73	5.87	2.12
14	1930.44	121.02	6.27	3.55
15	954.13	39.09	4.10	4.52
16	326.34	64.20	19.67	2.25
17	1097.40	53.40	4.87	4.05
18	1021.25	37.38	3.66	2.22
19	1500.33	82.06	5.47	4.37
20	2491.63	50.83	2.04	5.52
21	991.08	62.42	6.30	2.55
22	1419.06	96.21	6.78	6.52
23	1569.74	425.60	27.11	2.46
24	846.32	51.00	6.03	3.08
25	2112.42	68.64	3.25	5.28
26	2240.74	534.57	23.86	2.33
27	2128.77	66.62	3.13	3.83
28	1916.34	49.90	2.60	4.26
29	2017.23	57.77	2.86	No tumor
30	1236.79	85.88	6.94	No tumor

The mean and standard deviation values of breast density (percentage) were obtained.

**Fig 11:**
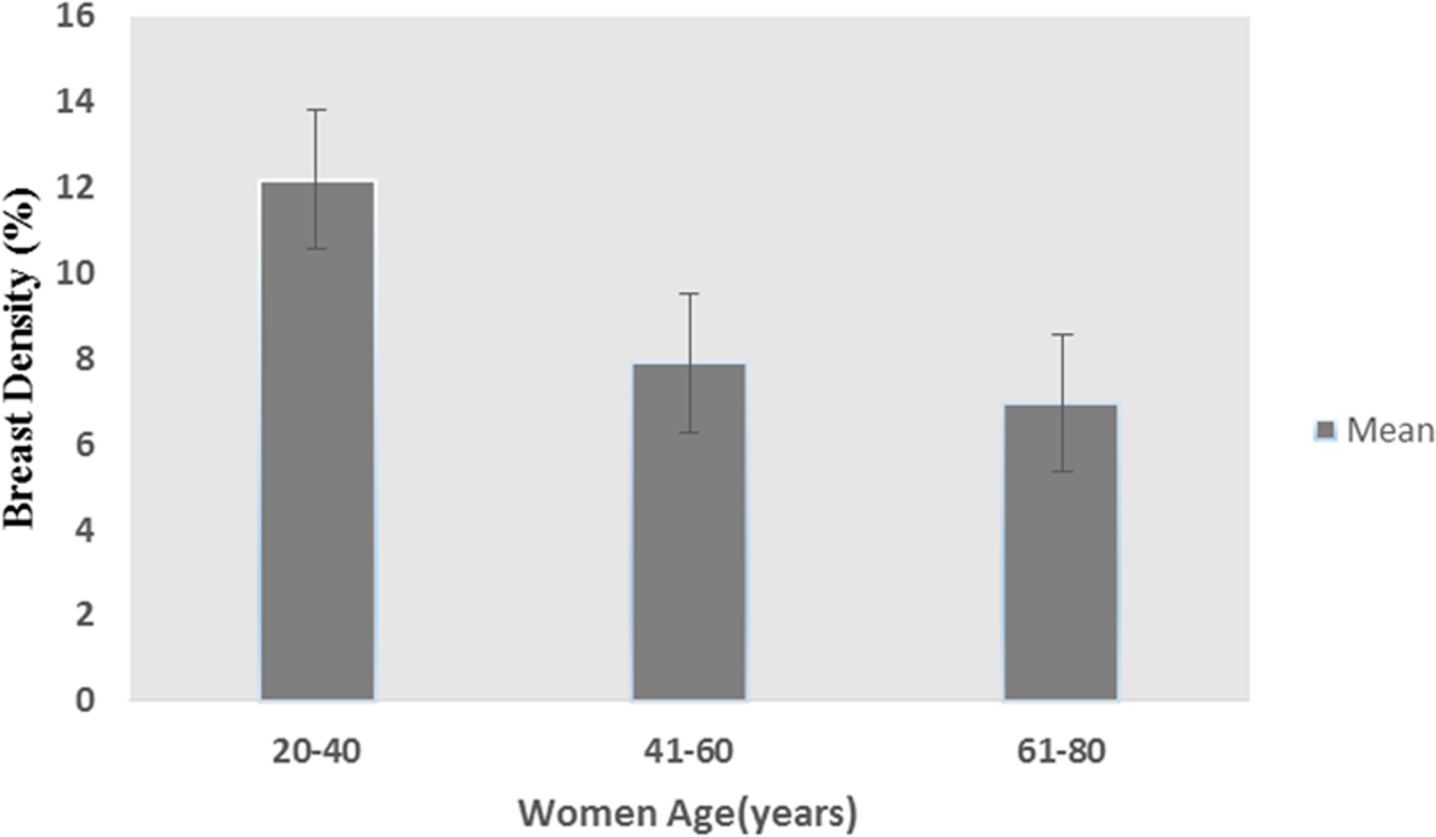
Bar graph of breast density vs age. The figure shows bar graph plot of breast density in different age groups. Error bars represent SD of breast density within each group.

Fig 11 shows the relationship of breast density with different age groups (20–40, 40–60, 60–80 years) for our cohort. Breast density shows an inverse relationship with age, which is in agreement with the reported studies[47]. The densities of the youngest age group are very different due to regional differences across the world [48]. Women with higher breast density are more prone to contralateral invasive diseases.

**Fig 12:**
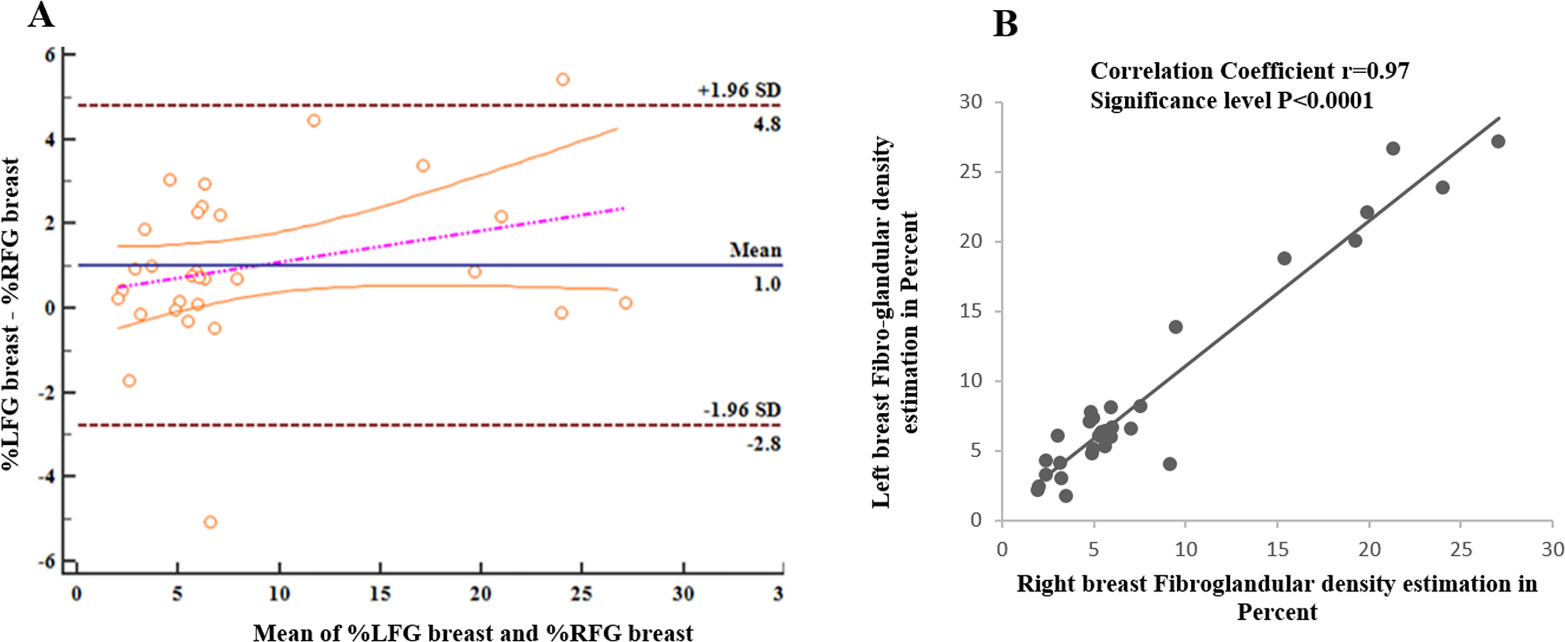
Bland-Altman and correlation plot. (A) and (B) show bland-Altman and correlation plots for left and right breast density of all the subjects.

The left and right breast densities were analyzed separately as shown in Table 6. In Fig 12A Bland-Altman plot shows the difference of the left and right breast density against their mean. The correlation coefficient of 0.97 was observed between left and right breast densities as shown in Fig 12B. Breast density shows an inverse relationship with age. Most of the subjects having age more than 40 years had low breast density (<10%), while others had greater than 10%. In the current study, tumor volume size is varied from 2.1cm3–7.08cm^3^ as shown in Table 5. The mean value of tumor volume was 3.8cm^3^ considering all patients.

**Table 6:** Total breast volume (cm^3^), FG tissue volume (cm^3^) and percentage of breast density for both left and right separately were calculated for each 30 patient datasets.

S. No	Total Right Breast volume (cm^3^)	Right Fibro-glandular (RFG) volume (cm^3^)	RFG Breast Density (%)	Total Left Breast volume (cm^3^)	Left Fibro-glandular (LFG) volume (cm^3^)	LFG Breast Density (%)
1	1009.57	20.42	2.02	977.06	24.07	2.46
2	885.04	176.15	19.90	894.37	197.47	22.07
3	736.21	176.81	24.02	708.45	169.35	23.90
4	817.87	48.24	5.90	783.33	47.11	6.01
5	762.06	71.86	9.43	657.97	91.38	13.89
6	768.52	41.76	5.43	867.20	54.80	6.32
7	858.45	64.54	7.52	780.86	64.25	8.23
8	682.62	33.83	4.96	699.73	35.85	5.12
9	860.79	42.84	4.98	857.37	63.39	7.39
10	830.06	44.06	5.31	839.73	50.92	6.06
11	246.68	38.02	15.41	183.75	34.51	18.78
12	707.59	64.60	9.13	863.97	35.03	4.05
13	905.65	43.43	4.79	810.98	57.30	7.07
14	980.14	47.29	4.82	950.29	73.73	7.76
15	624.06	18.97	3.04	330.07	20.13	6.10
16	158.83	30.54	19.23	167.51	33.65	20.09
17	492.67	24.10	4.89	604.73	29.30	4.85
18	510.75	16.09	3.15	510.50	21.29	4.17
19	757.46	42.51	5.61	742.87	39.56	5.32
20	1145.62	21.96	1.92	1346.01	28.87	2.14
21	525.74	31.35	5.96	465.34	31.07	6.68
22	687.88	48.34	7.03	731.17	47.88	6.55
23	839.80	227.23	27.06	729.95	198.37	27.18
24	397.11	22.35	5.63	449.21	28.65	6.38
25	1153.69	27.61	2.39	958.73	41.03	4.28
26	1177.88	250.62	21.28	1062.86	283.95	26.72
27	1086.62	34.71	3.19	1042.15	31.91	3.06
28	975.74	33.66	3.45	940.60	16.24	1.73
29	979.24	23.30	2.38	1037.98	34.47	3.32
30	672.68	39.94	5.94	564.10	45.94	8.14

The mean and standard deviation value of breast density (percentage) for left and right were obtained.

## Discussion

In this study, we have presented an automatic breast tissue segmentation approach using multi-parametric MRI images of breast tumor patients. This approach provided both outer and inner breast tissue segmentation, which enables automatic analysis of individual breast tissues. Breast tissue of patient data was successfully segmented out into FG, fatty and tumor tissues. The proposed unsupervised landmarks based method for outer segmentation works for all structural images. We have taken 30 patient dataset having large variations in breast shape, size and density. Proposed segmentation approaches are quite robust as it has been tested for different sizes, shapes, density and positions of the breast. The operation time for completing outer and inner segmentation of 40 slices with a matrix size of 512*512 was ~ 2 minutes on a personal computer with Intel(R) Xeon(R) 3.40 GHz CPU and 16 GB RAM.

The proposed approach enables automatic estimation of breast density. Breast density estimation plays a significant role in clinical aspects. This estimation could be used in follow-up studies (after chemotherapy) to detect small changes in breast density. This estimation can be taken into account for various studies such as epidemiological and parenchymal (morphological distribution pattern of the fibro-glandular tissue) etc. Women with higher breast density are more prone to contralateral invasive diseases. Longer follow-up of radiation may increase the risk of contralateral breast cancer [49]. Qualitative/quantitative analysis of tumor tissue is a key element for differentiating between benign and malignant. Moreover, machine learning based techniques for classification of the tumor can be automatized further using proposed segmentation approach. Volumetric analysis of tumor can also play a significant role in clinical application e.g. to detect a small change in tumor volume after neo-adjuvant chemotherapy (reduction in tumor size) or predicting the response of breast cancer. If tumor size does not vary with one neo-adjuvant chemotherapy, then a combination of drugs may be changed or can proceed with surgery.

As mentioned in the Result section standard segmentation methods were applied, but they failed to segment out breast region. Clustering method by Li et al. [27] was used to obtain initial segmentation. The gradient-based tracing algorithm was used for further refinement using seed points. Incorrect seed points can lead to failure of this existing method. Koenig et al.[50] used gray-level thresholding using the histogram. Intensity gradients were used for breast tissue boundary detection. This method is dependent upon the exact location of nipples and can only roughly segment the chest wall boundary. Automatic methods by Lei Wang et al. [22] was replicated and it worked well for most of the cases but found limitations in pectoral muscle separation using a vector-based connected component filter. Some parts of pectoral muscle were left over in the segmented tissue. Model-based methods use the complete breast as a template for performing segmentation [39]. Since the shape of breasts varies largely from subject to subject, a single universal template might not ensure robustness of the segmentation algorithms. SVM-based segmentation using 3-D Multi-parametric breast MRI by Yi Wang was reported for inner segmentation. However, with only non-contrast images, segmentation of FG and tumor/lesion tissue was less effective [35], particularly for tumor/lesions and FG tissues showing similar intensity on T_2_-W images. Atlas-based approach by Albert Gubern Merida et al.[34] was not suitable to segment FG tissue due to the high variability of the dense tissue.

In the proposed method, contrast and non-contrast multi-parametric MRI images were used for outer and inner segmentation. Proposed segmentation approach uses some landmark points particularly, P3’, P4 and P5. The points P4 and P5 specify the region where breast fat starts merging with body fat. Basically, these points are approximately starting points of the breast, so parts of the image below these two points are non-breast tissue. The thickness factor 1.5 for the selection point P4 and P5 (as described in data processing part) was based on empirical observation and it was validated by expert radiologists. In this study, we have used a middle slice for selection of P4 and P5 points and these points were applied for all the slices. Selection of these points was robust and provided accurate and fast segmentation. Selection of P4 and P5 on individual slices was less accurate, particularly in extreme slices. In the proposed method, for removal of pectoral muscle, neighboring pixel intensity information is incorporated to take a decision about a pixel. On structural images, breast fat tissue shows homogeneous intensity distribution and relatively higher intensity values compared to pectoral muscle. Our method also uses anatomical landmarks of breast MRI images to determine the points for spline fitting. Use of anatomical landmarks also makes proposed approach less sensitive to bias in signal intensity due to field inhomogeneity.

There is no fixed boundary marker in a breast, so the output of the breast outer segmentation has to be verified by radiologists and they may have a different opinion in the selection of breast boundary. As in the proposed method, we have tuned our parameters after consulting radiologists and the method gives satisfying results for all the datasets. We have also encountered few cases where breast tissues were slightly tilted/rotated. Our method worked well in these data sets. Additionally, we have also artificially rotated images up to 5 degrees. More than 5-degree rotation is difficult due to patient’s comfortable position. The proposed algorithm also worked well in such abnormal cases. For rotation of more than 5 degrees, the algorithm might need to be modified a little, because if the image is rotated more than 5 degrees, it may happen that during vertical screening from top to bottom, P4 or P5 point is nearby P1 or P2, which is not desired. In this case, we can start vertical screening for P4, P5 from the middle point of P1 and P2, which is P3. It will pick correct P4 and P5 for such cases. This could be one solution but to handle dataset with such non-ideal condition, a more efficient technique can be proposed.

Dice and Jaccard coefficients are measured to find the precision of segmentation. But these two methods are insensitive to volumetric under and over estimations. Jaccard Coefficient is numerically more sensitive to mismatch when there is reasonably strong overlap than Dice values. In the case of Outer Segmentation, Dice and Jaccard coefficients are measured using the pixels of the many slices, the sum-areas used to compute the metrics are very large, and at very large scales these measures can become distorted due to the sheer number of data points and give the appearance of over-performance [51].

The requirement of multiple images and accurate fat saturation for inner segmentation can be a limitation of the proposed method. However, in most of the breast MRI studies, these multiple types of images are routinely acquired. The proposed method is developed for MRI images acquired in the axial orientation only. Some studies do put saturation band to suppress signal from other body parts. In such cases, proposed method might require some changes. In the current study, most of the cases had a clear bright intensity boundary of fat tissue between FG and pectoral muscle. This boundary serves as stopping criteria during automatic pectoral muscle removal using vertical screening in our cohort. In the worst case scenario, if segmentation result is not satisfactory due to the unclear boundary and having a similar intensity of FG and pectoral tissue, then a hard limit can be included during vertical screening for pectoral muscle removal. For example, such limit can be derived from the maximum anatomical width of the pectoral muscle. We have observed that maximum width of pectoral muscle is ~10 mm in our data set. Images without a clear boundary can also be segmented well by using a hard limit. But, this proposed method has not been tested on a cohort with high mammographic density/ parenchymal fraction. Future studies shall be taken to include such data sets.

Proposed inner segmentation approach requires images with and without fat saturation. The accuracy of fat saturation should be reasonable. Based upon consultancy with experienced radiologists in the current study, mDIXON approach accurately suppressed fatty tissue. In the breast studies, having substantial B1 inhomogeneity, it might be difficult to achieve uniform fat saturation. In such studies, inner segmentation results might be compromised. The current approach for tumor/lesion tissue segmentation works only for contrast-enhanced tumors. In case proposed approach fails to separate tissues with strong background enhancement, additional features/parameters derived from DCE-MRI time curve can be included in inner segmentation approach to separate such tissues. Similarly, lesions without enhancement might have some features, which can be incorporated to segment such lesions. Future studies shall be taken to include such data sets. A partial volume might cause a small change in density estimation. This can be taken into account by actually estimating the fractional contribution of fat to voxels and multiplying the value of this fraction to each voxel before estimating density. Therefore, in future partial volume correction need to be taken care for accurate breast density estimation. In the current study, rigid motions were mitigated well. However, small (< 1mm) non-rigid motions were also visually noted while screening (using the slider) multiple MRI images of the same section in stack view. However, these motions were not mitigated by MATLAB inbuilt registration algorithm. Correction of these local elastic motions might further improve the accuracy. Correction of these motions requires a dedicated non-rigid motion correction approach, and further studies are needed to be carried out in this direction.

## Conclusions

In conclusion, we have presented breast tissue segmentation approach. Proposed automatic segmentation approach based upon multi-parametric MRI images worked well for breast tumor patients. It enables automatic segmentation of breast tissue from other body parts as well as provides segmentation of FG, fatty and tumor tissues. The proposed approach can be used for automatic computation of the volume of FG, fatty and tumor as well as breast density. Proposed segmentation approach is simple, fast and accurate.

## Supporting information

S1 Dataset DICOM images were used in the study (DOI: https://doi.org/10.6084/m9.figshare.5411392). (ZIP)
